# A Survey Exploring Gendered Racism Experienced by Junior Doctors Working in Psychiatry

**DOI:** 10.1192/bjo.2022.331

**Published:** 2022-06-20

**Authors:** Aicha Rais, Richard Burton, Adeel Rauf

**Affiliations:** Derbyshire Healthcare NHS Foundation Trust, Derbyshire, United Kingdom

## Abstract

**Aims:**

To measure rates of racism experienced and witnessed by Junior Doctors working at Derbyshire Healthcare NHS Foundation Trust.

**Methods:**

Surveys were sent out via e-mail and WhatsApp to all Junior Doctors from 22 November 2021 to 1 December 2021.

Questions asked about personal experiences of racism, witnessing racism to/from patients and/or staff whilst working in Derbyshire, knowledge of how to report incidents and if routinely reported. Doctor race and gender recorded.

**Results:**

88 Junior Doctors contacted. Response rate 55% (48 out of 88). 63% female, 35% male and 2% gender undisclosed. 37.5% White, 12.5% Black, 37.5% Asian, 6.3% Mixed-race, 4.2% Arab or other ethnic group and 2% Race undisclosed. 13% of doctors experienced racism from staff: 75% of the Black female population, 50% of the Black male population, 8% of the Asian female population and 17% of the Asian male population. 27% of doctors experienced racism from patients: 50% Black female population, 50% Black male population, 58% Asian female population, 16% Asian male population, 100% Mixed-race female population and 1 Race unspecified male. 13% of doctors witnessed racism from staff to other staff: 75% Black female population, 50% Black male population, 11% Asian female population and 16% Asian male population. 63% of doctors witnessed racism from patients towards staff: 75% Black female population, 50% Black male population, 67% Asian female population, 33% Asian male population, 100% of the Mixed-race population, 58% White female population, 83% of the White male population and by 1 male Race unspecified. Two reports of racism witnessed from staff towards patients. 50% of doctors do not know how to report racism. 54% of doctors would report racism if they knew how.

**Conclusion:**

Black, Asian, and Minority Ethnic (BAME) Junior Doctors are disproportionately affected by racism with female gender as an additional vulnerability. Mixed-race females, Asian females, and Black doctors gave highest reported experience of racism from patients. Black doctors gave a higher reported experience of racism from staff and reported witnessing the most racism from staff towards other staff. Mixed-race and White male doctors represent a high number of those that witness patients be racist towards staff. Additional support is required in encouraging allyship, confidence and ability to report racism.

